# Crystal structure of 3,6-bis­(2-chloro­phen­yl)-1,2,4,5-tetra­zine: the acaricide clofentezine

**DOI:** 10.1107/S1600536814021291

**Published:** 2014-09-30

**Authors:** Gihaeng Kang, Seonghwa Cho, Sangjin Lee, Tae Ho Kim

**Affiliations:** a Department of Chemistry and Research Institute of Natural Sciences, Gyeongsang National University, Jinju 660-701, Republic of Korea

**Keywords:** crystal structure, clofentezine, acaricide, π–π inter­actions

## Abstract

The whole molecule of the title compound, C_14_H_8_Cl_2_N_4_, is generated by inversion symmetry. The dihedral angle between the 2-chloro­phenyl ring and the tetra­zine ring is 47.65 (5)°. In the crystal, mol­ecules are linked by slipped parallel π–π inter­actions [centroid–centroid distance = 3.8199 (5), normal distance = 3.3127 (8), slippage 1.902 Å] forming columns along the *a*-axis direction.

## Related literature   

For information on the toxicity and acaricidal properties of the title compound, which is used in plant protection for the control of spider mites on a wide range of crops, see: Zhao *et al.* (1996 ▶); Ay & Ebru Kara (2011 ▶). For the structures of the *m*- and *p*-isomers, see: Infantes *et al.* (2003 ▶).
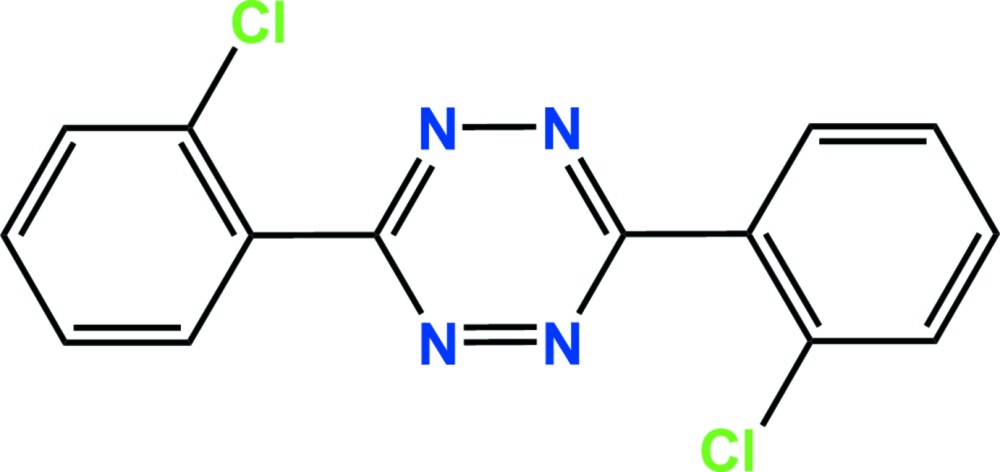


## Experimental   

### Crystal data  

C_14_H_8_Cl_2_N_4_
*M* *_r_* = 303.14
Monoclinic, 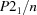

*a* = 3.8199 (4) Å
*b* = 14.0706 (16) Å
*c* = 12.1066 (15) Å
β = 97.715 (3)°
*V* = 644.82 (13) Å^3^
*Z* = 2
Mo *K*α radiation
μ = 0.50 mm^−1^
*T* = 173 K
0.45 × 0.09 × 0.06 mm

### Data collection  

Bruker APEXII CCD diffractometer
Absorption correction: multi-scan (*SADABS*; Bruker, 2009 ▶) *T*_min_ = 0.808, *T*_max_ = 0.971
4197 measured reflections
1456 independent reflections
1222 reflections with *I* > 2σ(*I*)
*R*_int_ = 0.031

### Refinement  

*R*[*F* ^2^ > 2σ(*F* ^2^)] = 0.038
*wR*(*F* ^2^) = 0.096
*S* = 1.07
1456 reflections
91 parameters
H-atom parameters constrained
Δρ_max_ = 0.44 e Å^−3^
Δρ_min_ = −0.21 e Å^−3^

### 

Data collection: *APEX2* (Bruker, 2009 ▶); cell refinement: *SAINT* (Bruker, 2009 ▶); data reduction: *SAINT*; program(s) used to solve structure: *SHELXTL* (Sheldrick, 2008 ▶); program(s) used to refine structure: *SHELXTL*; molecular graphics: *DIAMOND* (Brandenburg, 2010 ▶); software used to prepare material for publication: *SHELXTL*.

## Supplementary Material

Crystal structure: contains datablock(s) global, I. DOI: 10.1107/S1600536814021291/nk2226sup1.cif

Structure factors: contains datablock(s) I. DOI: 10.1107/S1600536814021291/nk2226Isup2.hkl

Supporting information file. DOI: 10.1107/S1600536814021291/nk2226Isup3.cml

**Fig. 1.**
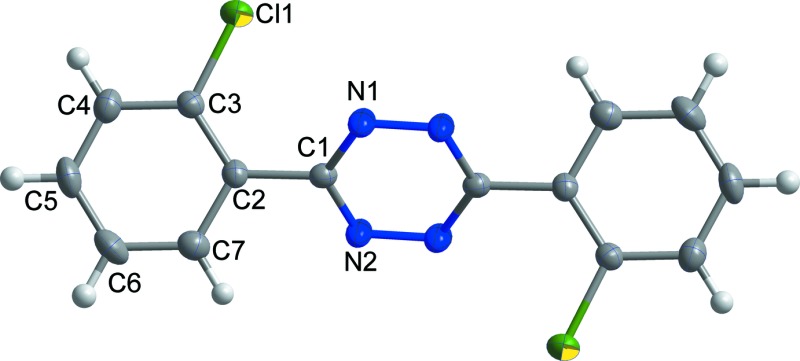
The asymmetric unit of the title compound with the atom-numbering scheme. Displacement ellipsoids are drawn at the 50% probability level. H atoms are shown as small spheres of arbitrary radius.

. DOI: 10.1107/S1600536814021291/nk2226fig1.tif
The asymmetric unit of the title compound with the atom-numbering scheme. Displacement ellipsoids are drawn at the 50% probability level. H atoms are shown as small spheres of arbitrary radius.

CCDC reference: 1026062

Additional supporting information:  crystallographic information; 3D view; checkCIF report

## supplementary crystallographic information

### S1. Comment

Clofentezine, C_14_H_8_Cl_2_N_4_, is an acaricide that is used in plant protection for the control of spider mites on a wide range of crops (Zhao *et al.*, 1996; Ay & Ebru Kara, 2011), and its crystal structure is reported herein. This molecule is located on a centre of symmetry, and a half molecule constitutes the asymmetric unit (Scheme 1, Fig. 1). The dihedral angle between a mean plane of the 2-chlorophenyl ring (r.m.s. deviation 0.0109 Å) and a mean plane of the tetrazine ring (r.m.s. deviation 0.0002 Å) is 47.65 (5)°. All bond lengths and bond angles are normal and comparable to those observed in the crystal structure of *m*- and *p*-isomers (Infantes *et al.*, 2003).

In the crystal structure, weak intermolecular face-to-face π···π interactions between the tetrazine ring systems [*Cg*1···*Cg*1^ii^, 3.8199 (5) Å] and the phenyl ring systems [*Cg*2···*Cg*2^ii^, 3.8199 (5) Å] link molecules in one-dimensional packing structure along [100] (*Cg*1 and *Cg*2 are the centroids of the N1···C1 and C2···C7 rings, respectively) [for symmetry codes: (ii), -*x* + 1, -*y* + 1, -*z* + 1].

### S2. Experimental

The title compound was purchased from the Dr Ehrenstorfer GmbH Company. Slow evaporation of a solution in CHCl_3_ gave single crystals suitable for X-ray analysis.

### S3. Refinement

All H-atoms were positioned geometrically and refined using a riding model with d(C—H) = 0.95 Å, *U*_iso_ = 1.2*U*_eq_(C) for aromatic C—H groups.

### Crystal data

**Table:**

C_14_H_8_Cl_2_N_4_	*F*(000) = 308
*M**_r_* = 303.14	*D*_x_ = 1.561 Mg m^−^^3^
Monoclinic, *P*2_1_/*n*	Mo *K*α radiation, λ = 0.71073 Å
Hall symbol: -P 2yn	Cell parameters from 1312 reflections
*a* = 3.8199 (4) Å	θ = 2.2–27.0°
*b* = 14.0706 (16) Å	µ = 0.50 mm^−^^1^
*c* = 12.1066 (15) Å	*T* = 173 K
β = 97.715 (3)°	Plate, red
*V* = 644.82 (13) Å^3^	0.45 × 0.09 × 0.06 mm
*Z* = 2	

### Data collection

**Table:**

Bruker APEXII CCD diffractometer	1456 independent reflections
Radiation source: fine-focus sealed tube	1222 reflections with *I* > 2σ(*I*)
Graphite monochromator	*R*_int_ = 0.031
φ and ω scans	θ_max_ = 27.4°, θ_min_ = 2.2°
Absorption correction: multi-scan (*SADABS*; Bruker, 2009)	*h* = −4→4
*T*_min_ = 0.808, *T*_max_ = 0.971	*k* = −14→18
4197 measured reflections	*l* = −15→15

### Refinement

**Table:**

Refinement on *F*^2^	Primary atom site location: structure-invariant direct methods
Least-squares matrix: full	Secondary atom site location: difference Fourier map
*R*[*F*^2^ > 2σ(*F*^2^)] = 0.038	Hydrogen site location: inferred from neighbouring sites
*wR*(*F*^2^) = 0.096	H-atom parameters constrained
*S* = 1.07	*w* = 1/[σ^2^(*F*_o_^2^) + (0.0377*P*)^2^ + 0.299*P*] where *P* = (*F*_o_^2^ + 2*F*_c_^2^)/3
1456 reflections	(Δ/σ)_max_ < 0.001
91 parameters	Δρ_max_ = 0.44 e Å^−^^3^
0 restraints	Δρ_min_ = −0.21 e Å^−^^3^

### Special details

**Table:**

Geometry. All e.s.d.'s (except the e.s.d. in the dihedral angle between two l.s. planes) are estimated using the full covariance matrix. The cell e.s.d.'s are taken into account individually in the estimation of e.s.d.'s in distances, angles and torsion angles; correlations between e.s.d.'s in cell parameters are only used when they are defined by crystal symmetry. An approximate (isotropic) treatment of cell e.s.d.'s is used for estimating e.s.d.'s involving l.s. planes.
Refinement. Refinement of *F*^2^ against ALL reflections. The weighted *R*-factor *wR* and goodness of fit *S* are based on *F*^2^, conventional *R*-factors *R* are based on *F*, with *F* set to zero for negative *F*^2^. The threshold expression of *F*^2^ > σ(*F*^2^) is used only for calculating *R*-factors(gt) *etc*. and is not relevant to the choice of reflections for refinement. *R*-factors based on *F*^2^ are statistically about twice as large as those based on *F*, and *R*- factors based on ALL data will be even larger.

### Fractional atomic coordinates and isotropic or equivalent isotropic displacement parameters (Å^2^)

**Table:**

	*x*	*y*	*z*	*U*_iso_*/*U*_eq_	
Cl1	0.72153 (14)	0.44093 (3)	0.68048 (4)	0.03196 (18)	
N1	1.1350 (5)	0.45432 (11)	0.91567 (14)	0.0288 (4)	
N2	0.8626 (5)	0.58937 (11)	0.98682 (13)	0.0281 (4)	
C1	0.9976 (5)	0.54235 (12)	0.90522 (16)	0.0221 (4)	
C2	1.0066 (5)	0.59412 (12)	0.79914 (15)	0.0219 (4)	
C3	0.9028 (5)	0.55442 (12)	0.69426 (16)	0.0229 (4)	
C4	0.9276 (5)	0.60556 (14)	0.59725 (17)	0.0310 (5)	
H4	0.8507	0.5782	0.5264	0.037*	
C5	1.0657 (6)	0.69676 (15)	0.60499 (19)	0.0355 (5)	
H5	1.0929	0.7310	0.5390	0.043*	
C6	1.1638 (6)	0.73814 (14)	0.7076 (2)	0.0349 (5)	
H6	1.2533	0.8012	0.7122	0.042*	
C7	1.1319 (5)	0.68778 (14)	0.80399 (18)	0.0296 (4)	
H7	1.1959	0.7172	0.8744	0.036*	

### Atomic displacement parameters (Å^2^)

**Table:**

	*U*^11^	*U*^22^	*U*^33^	*U*^12^	*U*^13^	*U*^23^
Cl1	0.0376 (3)	0.0258 (3)	0.0319 (3)	−0.0065 (2)	0.0026 (2)	−0.00350 (19)
N1	0.0405 (10)	0.0240 (8)	0.0224 (8)	0.0090 (7)	0.0064 (7)	0.0001 (6)
N2	0.0410 (10)	0.0225 (7)	0.0214 (8)	0.0082 (7)	0.0060 (7)	0.0006 (6)
C1	0.0238 (9)	0.0204 (8)	0.0220 (9)	0.0013 (7)	0.0022 (7)	−0.0021 (7)
C2	0.0219 (9)	0.0218 (8)	0.0228 (9)	0.0040 (7)	0.0053 (7)	0.0013 (7)
C3	0.0227 (9)	0.0212 (9)	0.0251 (10)	0.0013 (7)	0.0044 (8)	0.0007 (7)
C4	0.0340 (11)	0.0353 (11)	0.0238 (10)	0.0043 (9)	0.0046 (8)	0.0035 (8)
C5	0.0380 (12)	0.0340 (11)	0.0362 (12)	0.0042 (9)	0.0109 (10)	0.0170 (9)
C6	0.0326 (11)	0.0222 (9)	0.0506 (14)	−0.0005 (8)	0.0078 (10)	0.0084 (9)
C7	0.0297 (11)	0.0249 (9)	0.0338 (11)	0.0009 (8)	0.0029 (9)	−0.0006 (8)

### Geometric parameters (Å, º)

**Table:**

Cl1—C3	1.7397 (18)	C3—C4	1.391 (3)
N1—N2^i^	1.330 (2)	C4—C5	1.386 (3)
N1—C1	1.345 (2)	C4—H4	0.9500
N2—N1^i^	1.330 (2)	C5—C6	1.378 (3)
N2—C1	1.348 (2)	C5—H5	0.9500
C1—C2	1.481 (3)	C6—C7	1.385 (3)
C2—C3	1.395 (3)	C6—H6	0.9500
C2—C7	1.401 (3)	C7—H7	0.9500
			
N2^i^—N1—C1	117.74 (16)	C5—C4—H4	120.3
N1^i^—N2—C1	117.78 (16)	C3—C4—H4	120.3
N1—C1—N2	124.49 (17)	C6—C5—C4	120.48 (19)
N1—C1—C2	118.72 (16)	C6—C5—H5	119.8
N2—C1—C2	116.74 (16)	C4—C5—H5	119.8
C3—C2—C7	117.95 (17)	C5—C6—C7	119.98 (19)
C3—C2—C1	123.74 (16)	C5—C6—H6	120.0
C7—C2—C1	118.30 (17)	C7—C6—H6	120.0
C4—C3—C2	121.19 (17)	C6—C7—C2	120.95 (19)
C4—C3—Cl1	117.72 (15)	C6—C7—H7	119.5
C2—C3—Cl1	121.05 (14)	C2—C7—H7	119.5
C5—C4—C3	119.4 (2)		
			
N2^i^—N1—C1—N2	0.1 (3)	C7—C2—C3—Cl1	−176.46 (14)
N2^i^—N1—C1—C2	177.28 (17)	C1—C2—C3—Cl1	4.6 (3)
N1^i^—N2—C1—N1	−0.1 (3)	C2—C3—C4—C5	1.5 (3)
N1^i^—N2—C1—C2	−177.33 (17)	Cl1—C3—C4—C5	179.03 (15)
N1—C1—C2—C3	47.7 (3)	C3—C4—C5—C6	−2.7 (3)
N2—C1—C2—C3	−134.8 (2)	C4—C5—C6—C7	1.4 (3)
N1—C1—C2—C7	−131.16 (19)	C5—C6—C7—C2	1.2 (3)
N2—C1—C2—C7	46.3 (3)	C3—C2—C7—C6	−2.3 (3)
C7—C2—C3—C4	1.0 (3)	C1—C2—C7—C6	176.64 (18)
C1—C2—C3—C4	−177.93 (18)		

Symmetry code: (i) −*x*+2, −*y*+1, −*z*+2.
