# Supplementary figures and images for: Crystal structure of 3,6-bis­(2-chloro­phen­yl)-1,2,4,5-tetra­zine: the acaricide clofentezine
Source: Acta Crystallogr Sect E Struct Rep Online. 2014 Sep 30;70(Pt 10):o1135. doi: 10.1107/S1600536814021291 (PMC4257200; doi:10.1107/S1600536814021291)

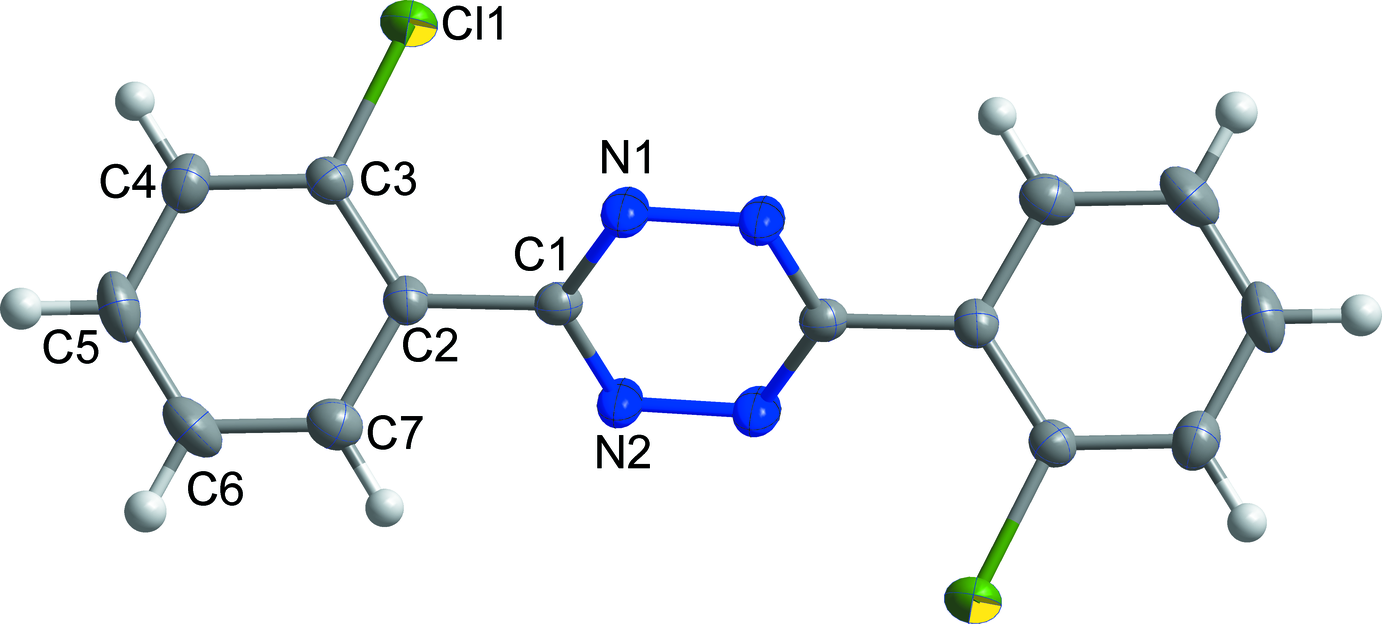

Supplement: Supplementary file 4 [file e-70-o1135-fig1.tif]
